# Functional, anatomical and diffusion tensor MRI study of radiology expertise

**DOI:** 10.1371/journal.pone.0231900

**Published:** 2020-04-27

**Authors:** David J. Ouellette, Eric Van Staalduinen, Syed H. Hussaini, Sindhuja T. Govindarajan, Patricia Stefancin, Dan-Ling Hsu, Timothy Q. Duong

**Affiliations:** 1 Biomedical Engineering, Stony Brook University, Stony Brook, New York, United States of America; 2 Radiology, Stony Brook University, Stony Brook, New York, United States of America; Xidian University, CHINA

## Abstract

**Background:**

Repeated practice to acquire expertise could result in the structural and functional changes in relevant brain circuits as a result of long-term potentiation, neurogenesis, glial genesis, and remodeling.

**Purpose:**

The goal of this study is to use task fMRI to study the brain of expert radiologists performing a diagnosis task where a series of medical images were presented during fMRI acquisition for 12s and participants were asked to choose a diagnosis. Structural and diffusion-tensor MRI were also acquired.

**Methods:**

Radiologists (N = 12, 11M, 38.2±10.3 years old) and non-radiologists (N = 17, 15M, 30.6±5.5 years old) were recruited with informed consent. Medical images were presented for 12 s and three multiple choices were displayed and the participants were asked to choose a diagnosis. fMRI, structural and diffusion-tensor MRI were acquired. fMRI analysis used FSL to determine differences in fMRI responses between groups. Voxel-wise analysis was performed to determine if subcortical volume, cortical thickness and fractional anisotropy differed between groups. Correction for multiple comparisons used false discovery rate.

**Results:**

Radiologists showed overall lower task-related brain activation than non-radiologists. Radiologists showed significantly lower activation in the left lateral occipital cortex, left superior parietal lobule, occipital pole, right superior frontal and precentral gyri, lingual gyrus, and the left intraparietal sulcus (p<0.05). There were no significant differences between groups in cortical thickness, subcortical volume and fractional anisotropy (p>0.05).

**Conclusions:**

Radiologists and non-radiologists had no significant difference in structural metrics. However, in diagnosis tasks, radiologists showed markedly lower task-related brain activations overall as well as a number of high-order visual and non-visual brain regions than non-radiologists. Some brain circuits appear to be uniquely associated with differential-diagnosis paradigm expertise that are not involved in simpler object-recognition cases. Improved understanding of the brain circuitry involved in acquisition of expertise might be used to design optimal training paradigms.

## Introduction

Expertise is the ability to produce a high level of performance consistently in a specific domain [1]. Through repeated practice, experts are able to routinely produce remarkable results in their chosen field. The amount of practice required to accomplish such feats results in the structural and functional reorganization of the relevant brain regions as a result of long-term potentiation, neurogenesis, glial genesis, and remodeling of different cellular and vascular components [2].

MRI has been used extensively to characterize structural changes to the brain associated with expertise. T1-weighted MRI showed gray and white matter volume differences between experts and novices in such domains as chess [3, 4] and musical performance [5, 6]. Diffusion tensor imaging (DTI) showed microstructural changes in the white matter tracts of motor-related experts, such as gymnasts [7, 8] and golfers [9]. Functional MRI (fMRI) has been used to detect task-specific differences in brain activation patterns between experts and novices in domains ranging from memory games to soccer [10–12]. MRI helps to uncover functional and structural adaptations of key systems associated with expert performance.

In regards to radiology expertise, there have been a few fMRI studies involving simple recognition tasks of medical images by expert radiologists [13–17]. There have been no task fMRI studies of radiologists while solving more difficult diagnoses or differentiating between two probable pathologies. Cognitive problem-solving research shows that experts typically use different strategies for simple problems than they do for difficult problems [18]. A review of cognitive research in medicine found that the same holds true for physicians generating diagnoses: an “easy” case is a matter of pattern recognition, while more difficult cases require a “hypothetico-deductive” approach, in which doctors form an initial hypotheses and test it against the available data [19, 20]. In radiologists, Melo et. al found that identifying abnormalities on chest x-rays activated the same brain regions as identifying animal shapes, suggesting that radiologists use the same process for categorizing medical images as non-radiologists use for common everyday objects [16]. This is further supported by Bilalic et. al who showed that radiologists engage the fusiform face area when discriminating between medical and non-medical images [13]. It is also supported by radiologists themselves, who report that with simple cases where no probable differential diagnosis exists, diagnosis becomes akin to object recognition: an intuitive and near-instantaneous task [16]. However, in difficult cases, radiologists could not employ a simple recognition strategy. Moreover, there have been no published structural and diffusion tensor MRI studies associated with radiology expertise.

The main goal of this study is to use task fMRI to study the brain of expert radiologists while performing a difficult diagnosis task in which a series of medical images with a brief patient history are presented during fMRI acquisition for 12s and participants are asked to choose a diagnosis. Comparisons were made with age- and education-matched non-radiologists. The images were chosen by qualified radiology experts to be difficult or ambiguous to interpret, thus forcing an alternative to the pattern recognition strategy used in previous fMRI studies of radiologists. As mentioned above, the literature has so far focused on the immediate, “gestalt” visual processing of radiologists. We predict that our task will not only activate brain areas involved in such gestalt visual processing and pattern recognition as has been reported in previous fMRI studies of radiology expertise [13, 14, 16], but our task will also activate brain areas involved in hypothetico-deductive problem-solving. This work will further our understanding of the underlying executive functions, spatial processing of working memory, and problem solving associated with radiology expertise. In addition, we also collected anatomical and diffusion-tensor MRI, and analyzed for differences in cortical thickness, subcortical gray matter volume, and fractional anisotropy in white matter between radiologists and non-radiologists.

## Methods

### Participants

This study was approved by the Stony Brook Institutional Review Board, approval #1077893. All participants provided written, informed consent. Participant demographics are provided in Table 1. Participants were recruited from the University Hospital and were split into two groups: radiologists and non-radiologists. Radiologists (N = 12, 11M, age = 38.2±10.3) were defined as participants that have passed the American Board of Radiology Core Exam, including attending radiologists, radiology fellows and 4th year radiology residents. Non-radiologists (N = 17, 15M, age = 30.6±5.5) included non-radiologist physicians and researchers with PhD or MD degrees in fields that did not involve medical imaging, 1st year radiology residents, interns, and 4th year medical students matched with radiologists for age and education. There was however a significant difference in age between radiologist and non-radiologist groups, which was addressed by adding age as a nuisance regressor in the analysis of group differences.

**Table 1:** Subject demographics.

Experts	N	Age	Non-experts	Novice	Age
Radiologist attendings	4M/0F	50.8 ± 5.6	Attendings/researchers	2M/1F	40.3 ± 5.8
Fellows	3M/1F	34.5 ± 4.5	Non-radiologist fellows	0	
4^th^ yr radiology residents	4M/0F	29.3 ± 1.0	1^st^ yr radiology residents	3M/1F	30.5±3.1
			4^th^ yr medical students	10M/1F	27.4±1.4

Our behavioral performance scores of attending radiologists and 4^th^ year residents were not statistically different, justifying combining the two groups. In addition, previous studies of resident and attending radiologists have shown comparable diagnosis accuracy [21, 22].

### fMRI tasks

**Fig 1:**
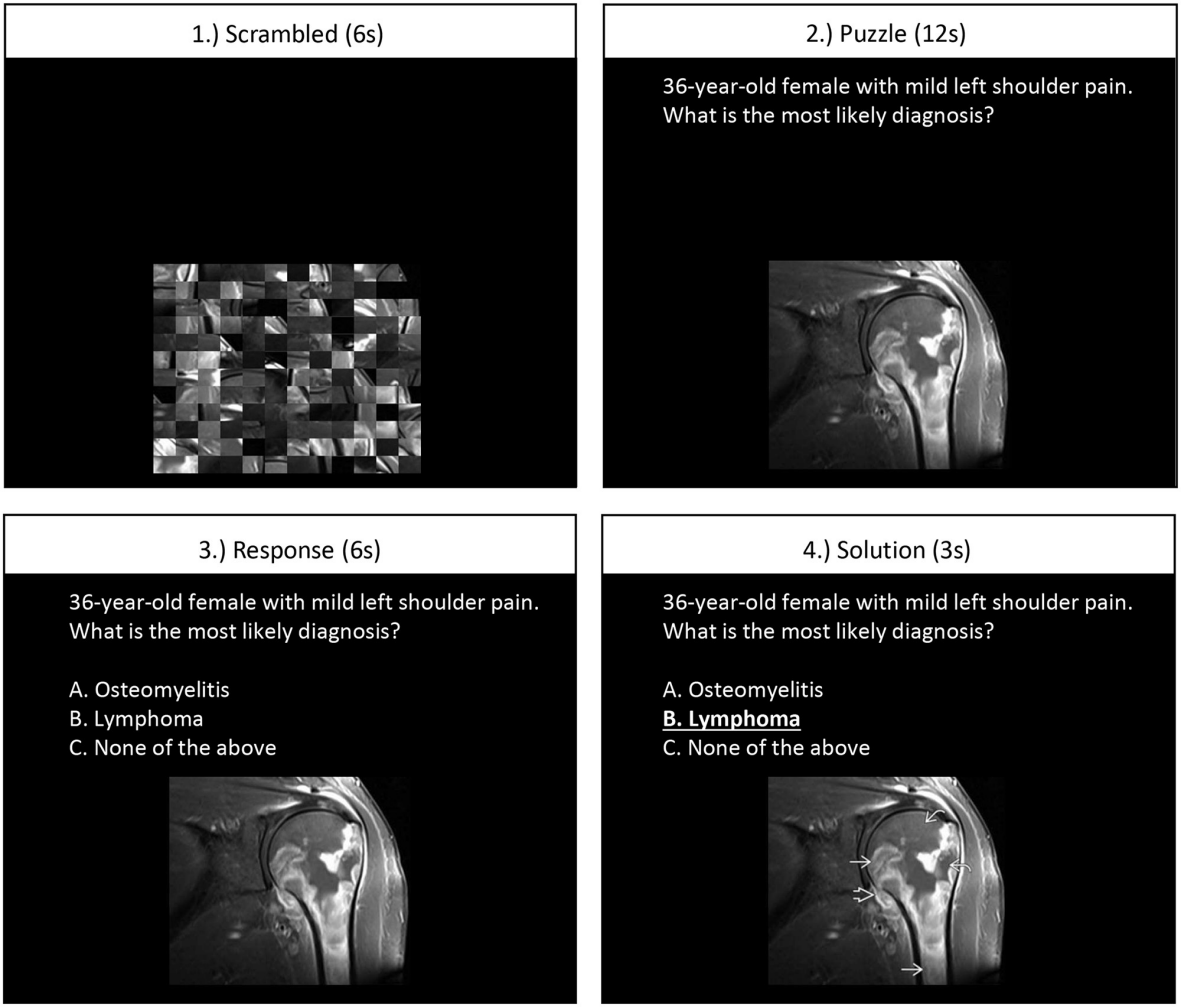
fMRI task paradigm.

The fMRI task paradigm is summarized in Fig 1. The paradigm was explained to subjects outside of the MRI scanner, and a short three-question training paradigm was run with the subject inside of the scanner prior to data acquisition. Subjects were first shown a scrambled radiological image. In the “puzzle” phase, subjects were instructed to examine a medical image with some patient history for 12 seconds and search for possible causes for the symptoms or other abnormalities. Finally, in the “solution” phase, subjects were asked to choose a diagnosis by pressing a button, and then shown the correct solution. The responses to each question was recorded with E-Prime software (Psychology Software Tools, Pittsburgh, PA). The images and questions were chosen by 2^nd^ year radiology residents from a database of board exam practice questions. The images were selected such that each would have at least two probable diagnoses that would require expertise to differentiate. All questions were based in abdominal, musculoskeletal, and neurological imaging using x-ray, CT, and MRI modalities. Each run consisted of 20 question trials lasting a total of 9 minutes.

### MRI acquisition

MRI experiments were performed at the University Hospital (3T Siemens Biograph mMR). Sagittal T1-weighted structural MRI were acquired using 3D MPRAGE with a repetition time (TR) = 2300 ms, echo time (TE) = 3.24 ms, field of view (FOV) = 19.2x22.3x22.3 cm, matrix = 192x256x256, voxel size = 0.9x0.9x0.9 mm. BOLD fMRI was acquired with TR = 2000 ms, TE = 30 ms, FOV = 19.5x19.5x10.2 cm, matrix = 78x78x31, voxel size = 2.5x2.5x3.3 mm. DTI was acquired with b = 0 and b = 1000 (30 directions), matrix = 96x96x65, voxel size = 2x2x2 mm, FOV = 19.2x19.2x13 cm. In addition, for estimation of the susceptibility-induced off-resonance field, three EPI volumes with the opposite phase encoding direction were acquired. Field estimations were calculated using FSL’s Topup tool [23, 24].

### fMRI analysis

All fMRI data processing was performed using the FMRIB Software Library (FSL) [25, 26]. Individual fMRI images were motion-corrected with a rigid-body registration to the middle volume using MCFLIRT [27]. Images were smoothed with a Gaussian spatial filter with a full width half maximum (FWHM) of 6 mm. One explanatory variable (EV) was defined for each phase of the task paradigm by convolving a square wave with a gamma function. The temporal derivatives of each EV were also added to the General Linear Model to account for small phase shifts in the data. The six motion parameters estimated by MCFLIRT, along with their squares, derivatives, and squares of their derivatives, were included as nuisance regressors to remove the effects of motion. Age of the participants was also added as a nuisance regressor, as the mean age of the radiologists tended to be higher than non-radiologists. Brain activation patterns during the puzzle phase were contrasted with activation upon viewing the scrambled image in order to remove brain activity that was due to simple visual stimulation. Comparisons were then made between radiologists and non-radiologists with a mixed effects model in FSL [26]. Significant activation was defined as a z-score greater than 2.6 and a cluster probability p-value of less than 0.05.

### Structural analysis

T1 images were segmented and parcellated using Freesurfer [28]. Cortical thickness maps were created and mapped onto an average template created from the study population using the standard Freesurfer workflow. Cortical thickness was compared between radiologists and non-radiologists on a vertexwise basis with a two-tailed t-test with the False Discovery Rate held to 0.05 to correct for multiple comparisons. Subcortical gray matter structures were also segmented using Freesurfer. The volumes of subcortical structures, including the caudate, putamen, thalamus, hippocampus, and globus pallidus, were normalized by total intracranial volume and compared between groups with a two-tailed t-test.

### DTI analysis

DTI images were linearly registered to each subject’s T1 scan and the result was nonlinearly registered into MNI space using FSL’s FLIRT and FNIRT tools, respectively [27]. Voxel-wise analysis was performed on fractional anisotropy (FA) images with nonparametric permutation inference through FSL’s randomise tool, using 5000 permutations and threshold-free cluster enhancement for multiple comparison correction [29].

## Results

**Fig 2:**
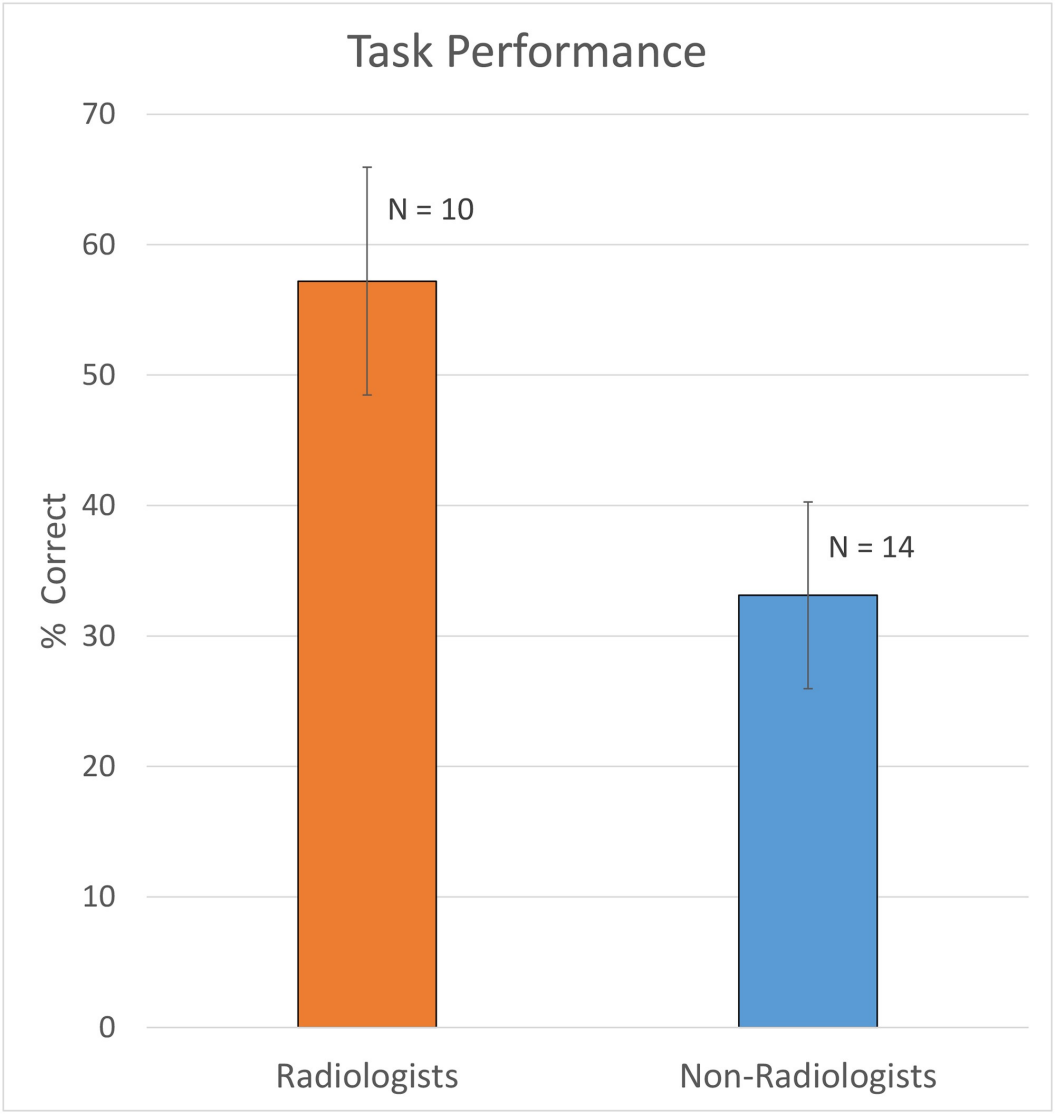
Radiologists performed significantly better than non-radiologists, whose results were close to chance. The sample sizes were smaller than fMRI sample sizes due to technical failure in recording the responses.

Behavioral data are shown in Fig 2. Radiologists chose the correct diagnosis 57 ± 9% of the time, significantly more accurate than random guessing on the 3-option multiple choices (p<0.001). Non-radiologists chose the correct diagnosis 33 ± 7% of the time, statistically no different from random guessing (p = 0.9). Note that the task was intentionally designed to be challenging so that radiologists could not get the right answers all the time for the given time allotted to make the decision. There was no significant difference in accuracy between 4^th^ year residents and the attending radiologists.

**Fig 3:**
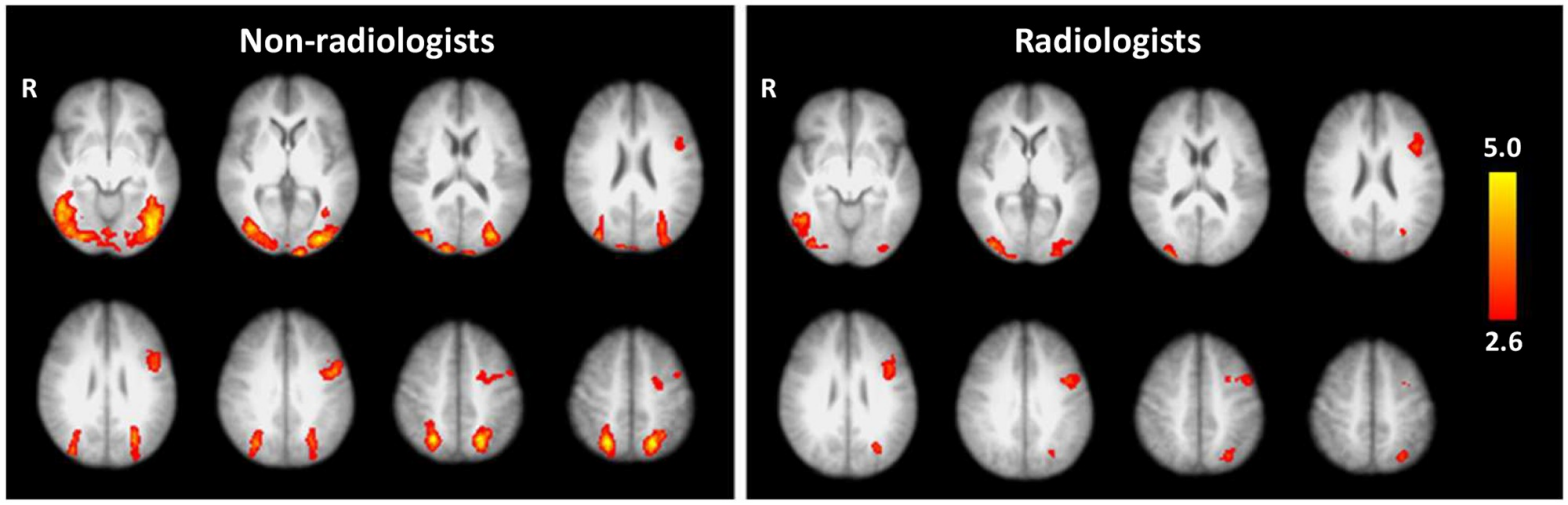
Mean activation during the puzzle phase was spatially similar in radiologists and non-radiologists, with non-radiologists showing bilateral task-related activation and radiologists showing lower activation overall.

Radiologists showed overall lower task-related brain activation than non-radiologists (Fig 3). In fact, there was no brain region where radiologists had higher activation. These results suggest that experts used less energy in performing the tasks compared to non-radiologists.

**Fig 4:**
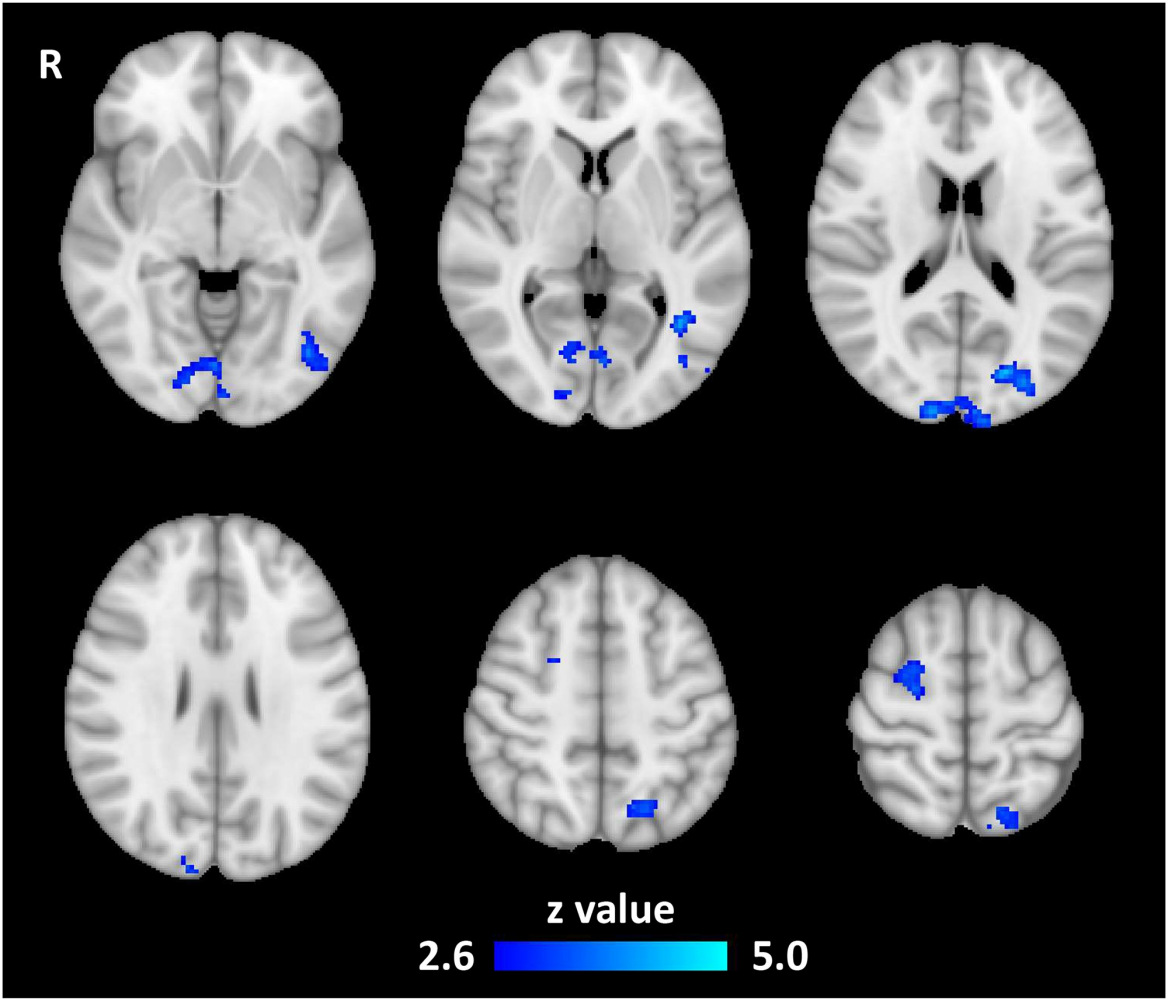
Radiology experts had lower task-related activation in visual and spatial processing areas.

When compared using a mixed-effects model, radiologists showed significantly lower activation in several areas: the lateral occipital cortex (LOC, inferior and superior divisions), the left superior parietal lobule (SPL), the right superior frontal gyrus (SFG), the right precentral gyrus, the lingual gyrus (LG), and the left intraparietal sulcus (IPS). (Fig 4, Table 2).

**Table 2:** Areas where radiologists had lower activation than non-radiologists.

Region	System	Z-Max MNI Coordinates	Size (voxels)	Max Z-Score
Occipital Pole (V2), Lingual Gyrus	Visual	12, -96, 22	635	5.02
L. Superior Parietal Lobule		-18, -66, 54	292	4.22
L. Inferior LOC		-38, -58, 4	278	4.14
L. Intraparietal sulcus		-26, -84, 14	268	4.73
R. Superior Frontal, R. Precentral	Somatosensory	24, -10, 62	263	3.94

### LOC & SPL

Radiologists had lower task-related activation in the lateral occipital cortex while viewing medical images than non-radiologists. The LOC is associated with object and shape recognition [30], and our result agrees with Harley et al.’s finding that disengagement of the LOC in radiologists in a lesion-location task [14]. The superior parietal lobule, another visual region, also showed lower task-related activation in radiologists. The SPL is typically activated during spatial cognition tasks such as mental rotation of 3D shapes [31] and other manipulations of spatial information in working memory.

### IPS

Radiologists had lower activation than non-radiologists in a significant cluster centered on the deep intraparietal sulcus (IPS), at the junction of the superior and inferior parietal lobules. The IPS is implicated in a wide variety of functions. Like the superior parietal lobule, the IPS has also been associated with manipulation of information in working memory, as well as directing visual attention and mediating saccades [32, 33]. More recently, it has been pinpointed as a crucial component in response inhibition [34], a key executive function.

### LG

The lingual gyrus has been shown to be involved in visual memory, during both the initial encoding and subsequent recall of complex images [30]. Previous fMRI studies have also shown that the lingual gyrus is associated with visual attention, including searching for a specific object in a crowded visual field [35]. While we found that the lingual gyrus was active in the puzzle versus scramble condition in non-radiologists, there was no significant activation in radiologists.

### Pre-central and superior frontal gyri

There was also a difference in task-related activation in radiologists in areas not traditionally associated with vision. One significant cluster covered parts of the right pre -central and superior frontal gyri in a region most commonly associated with hand movement [36, 37]. However, since all subjects had the response buttons on their right hands, unilateral activation differences in the right hemisphere are not likely caused by motor effects. The cluster overlaps the anterior dorsolateral region of the right SFG, which is consistent with the supplementary motor area (SMA) [30]. Recently it has been demonstrated that the SMA is not purely a motor region: fMRI experiments have shown that it can be activated by working memory, language, and sensory tasks [31, 32].

### Volumetric and DTI results

There were no significant differences in cortical thickness between radiologists and non-radiologists, either by vertexwise analysis or by regional averages using Freesurfer’s cortical atlas. There were no significant differences between groups in total grey and white matter volumes. There were no significant differences subcortical gray matter structures. Voxelwise analysis of FA in the white matter tracts showed no difference between radiologists and non-radiologists.

## Discussion

The most striking observation is that radiologists showed distinctly lower task-related brain activations overall than non-radiologists. These findings are consistent with previous fMRI studies of other expertise domains (music [38], golf [39], and race car driving [40]) which found that practice reduces the extent of activation during tasks related to the expertise domain. Glucose metabolism has also been found to be reduced in task-related areas of the brain after training [41]. Thus the reduced fMRI activations in radiologists may be a result of increased neural efficiency due to training.

There were no significant volumetric or fractional anisotropy differences in the brains of radiologists and non-radiologists. This is somewhat unexpected, as training-induced changes in brain morphology are well-established in other domains [42, 43]. Because of the heterogeneity of these parameters even within groups of healthy controls, it is possible such differences do exist between our study populations, but were unable to reach significance due to our sample size or relatively large age range.

As designed, the diagnosis task in this study evoked markedly different activation patterns than object recognition tasks from previous studies. Melo et al. compared lesion location to object recognition explicitly, showing that fast visual recognition of lesions in chest x-rays activated the same brain regions as an object-naming task. The finding suggests that the first step in a radiologists forming a diagnosis is akin to a visual recognition task rather than one involving higher cognitive functions [13]. In general, our fMRI diagnosis tasks activated the same regions as Melo et al, although it is less extensive in the visual areas and activation in the inferior frontal gyrus was limited to the left hemisphere. In addition, we found activation in areas that are not engaged by the object-recognition task of Melo et al., including the precentral gyrus and the middle frontal gyrus (Fig 3). These differences are likely because Melo et al. focused on the radiologist’s immediate reaction to the stimulus, and did not present ambiguous images that required careful consideration. We believe our differential-diagnosis paradigm engaged areas of the brain critical to radiology expertise that are not involved in simpler object-recognition cases.

As expected with visual experts, visuospatial areas had significantly different task-related activation between radiologists and non-radiologists. Radiologists had lower activation in the lateral occipital cortices, the occipital pole in the area of V2, the left superior parietal lobule and IPS, and the lingual gyrus. The result in the LOC replicates the result of a lung-nodule recognition study by Harley et al., which also compared activations between first and fourth year radiology residents [11]. Harley et al. also found increased activation in the fusiform face area (FFA) of fourth year residents. While our study replicated the finding in the lateral occipital cortex, elsewhere our activation patterns were more extensive than those of Harley et al. This is likely due to differences in task paradigms.

The SPL and IPS are spatial processing regions, which have a key function in common: manipulation of visual information in working memory. Manipulation of working memory is a common operation in cognitive expertise domains and is frequently cited as an important aspect of how experts are able to process large amounts of information quickly [44, 45]. The IPS is responsible for some executive functions as well, including inhibition and visual attention [46]. These executive functions may play a role in the group differences detected with our diagnosis task, but a more specific task-based study would be needed to confirm.

Lower task-based activation in the SPL and IPS may be due in part to the relative ease and speed with which radiologists direct their attention to the relevant areas of a medical image, and how quickly they are able to analyze the visual data they find there. The group difference in the activation of the lingual gyrus supports this claim. The LG is activated in non-radiologists, but not significantly activated by the task in radiologists. This could be due to the LG’s role in visual search and locating objects in a crowded visual field [35]. Eye-tracking studies have shown that radiologists do not perform a visual search in the same sense that non-radiologists do; radiologists are able to quickly direct their attention to the abnormal area of an image, ignoring the irrelevant anatomy [12]. This enhanced perceptive ability may be the reason that the lingual gyrus is not activated by the diagnosis task in experts.

Trained radiologists had lower activation in the lateral SFG in the area of the SMA, which, in addition to motor activity, is associated with working memory and language. More relevant to radiology expertise, the SMA and lateral SFG in general have been shown to be activated by visuospatial working memory tasks [47]. The common theme of lowered activation in radiologists in regions associated with working memory while viewing medical images suggests that working memory is either more efficient in radiologists, or is simply less important to their image analysis than it is to non-radiologists. Either way, it denotes a crucial component of radiology expertise.

One possible limitation of this study is the sample size, which may explain the lack of statistically significant differences in FA and cortical thickness which have been reported in other studies of expertise. In addition, our fMRI task was designed to simulate the clinical diagnosis process for radiologists. This allowed us to observe widespread functional differences in the brains of radiologists and non-radiologists. However, this has left more focused analysis of the individual aspects of radiology expertise (working memory, attention, integration of information) up to future studies. Another limitation is the lack of a task-free fMRI acquisition, or “resting state” fMRI. Such data would provide connectivity information between the different brain regions implicated in this study, and would enable for the correction of the task-based fMRI due to low-frequency fluctuations [48], which have been shown to be altered in some domains of expertise [49].

In conclusion, these findings suggest that radiology expertise acquisition involves a large, widespread and integrated functional network This work will further our understanding of the underlying executive functions, spatial processing of working memory, and problem solving associated with radiology expertise. Improved understanding of the brain circuitry involved in acquisition of expertise might be used to design optimal training paradigms and to evaluate acquisition of expertise.
